# Insights on Cytochrome P450 Enzymes and Inhibitors Obtained Through QSAR Studies

**DOI:** 10.3390/molecules17089283

**Published:** 2012-08-03

**Authors:** Jayalakshmi Sridhar, Jiawang Liu, Maryam Foroozesh, Cheryl L. Klein Stevens

**Affiliations:** 1 Department of Chemistry, Xavier University of Louisiana, 1 Drexel Dr., New Orleans, LA 70125, USA; 2 Ogden College of Science & Engineering, Western Kentucky University, 1906 College Heights Blvd., Bowling Green, KY 42101, USA

**Keywords:** 3D-QSAR, SAR, binding/active site, CoMFA, pharmacophore

## Abstract

The cytochrome P450 (CYP) superfamily of heme enzymes play an important role in the metabolism of a large number of endogenous and exogenous compounds, including most of the drugs currently on the market. Inhibitors of CYP enzymes have important roles in the treatment of several disease conditions such as numerous cancers and fungal infections in addition to their critical role in drug-drug interactions. Structure activity relationships (SAR), and three-dimensional quantitative structure activity relationships (3D-QSAR) represent important tools in understanding the interactions of the inhibitors with the active sites of the CYP enzymes. A comprehensive account of the QSAR studies on the major human CYPs 1A1, 1A2, 1B1, 2A6, 2B6, 2C9, 2C19, 2D6, 2E1, 3A4 and a few other CYPs are detailed in this review which will provide us with an insight into the individual/common characteristics of the active sites of these enzymes and the enzyme-inhibitor interactions.

## 1. Introduction

The cytochrome P450 (CYP) enzymes are heme-thiolate enzymes involved in the metabolism of a large number of exogenous molecules (natural products, drugs, and environmental carcinogens) and endogenous compounds such as hormones. The human CYPs are encoded by 57 genes [1] and are classified into four classes. The Class I and Class II CYPs (majority of the CYPs) are versatile monooxygenases catalyzing a large number of reactions such as conversion of alkenes to epoxides, alkanes to alcohols, arenes to phenols, and oxidation of sulfides. CYP enzymes belonging to the 1, 2 and 3 CYP families have been found in healthy and cancerous hepatic tissues [2,3]. The metabolism of carcinogens, pro-carcinogens, and chemotherapeutics by CYPs gives them an indisputable role in the cancer prevention and treatment strategies. CYPs 1B1 and 2W1 are indeed expressed specifically in tumors [4,5,6,7,8,9]. Numerous studies have implicated a role for CYPs in tumor formation and development [4,5,10,11,12,13,14]. Inhibition of CYPs is a widely pursued area of research for the treatment and prevention of cancer [15,16]. The CYP enzymes can be targeted by small molecules as delineated in three strategies: (1) inhibit the enzyme through competitive inhibitors; (2) inhibit the enzyme through mechanism-based inhibitors that result in the modification of the enzyme; and (3) design prodrugs that are activated by the CYPs. Intense effort is ongoing by many research groups to find specific and potent CYP inhibitors for the individual members of the CYP superfamily. 

Understanding the key structural features of the inhibitors responsible for their inhibition potency has been essential for CYP inhibitor design and development. Computational methods such as docking studies, and quantitative structure activity studies (QSAR) have been extensively employed toward this end as outlined in various review articles [17,18,19,20,21,22,23,24]. This review will provide the summary of the studies covered in these review articles and also the most recent developments including the use of machine learning methods, neural network analysis and multidimensional QSAR analysis. QSAR methods have evolved over the last two decades from a linear relationships method (Free Wilson method [25] and Hansch analysis [26,27]) to multiple linear regression methods using grid-based 3D-QSAR approaches (CoMFA) [28] and the more statistical intensive methods that include neural networks [29], subsequent variants [30,31], and decision trees [32,33]. QSAR analysis employs statistical methods by which biological activities (most often expressed by logarithms of equipotent molar activities) are related with structural elements (Free Wilson analysis), physicochemical properties (Hansch analysis), or fields (3D-QSAR). QSAR analyses can employ many levels of hierarchy in their methods as in 2D-QSAR, 3D-QSAR, and multidimensional QSAR methods (4D, 5D and 6D QSAR’s). The 2D-QSAR methodologies encompass fragment-based methods such as fragment-similarity-based QSAR (FS-QSAR), fragment-based QSAR (FB-QSAR), and Hologram QSAR (HQSAR) which employ traditional methods that use 2D molecular substituents or fragments and their physicochemical properties to perform quantitative predictions. The 3D-QSAR methods use the 3D conformers of the molecule that were introduced by Cramer called comparative molecular field analysis (CoMFA). Further developments on CoMFA lead to other methods such as CoMSIA, SOMFA, and CoMMA. In general, the 3D-QSAR methods were grid-based methods that are either alignment-dependent (CoMFA, CoMSIA, SOMFA) or alignment-independent (AMSP, CoMMA, WHIM). Multidimensional (nD) QSAR methods are extensions of the 3D-QSAR methods incorporating additional properties (dimensions) to overcome the limitations of 3D-QSAR. Most of these methods have been utilized by various groups for structure activity studies on the substrates and inhibitors of CYPs.

Two approaches to QSAR methods used for predicting the metabolism of substrates and inhibitors by CYPs have been detailed in the literature. The first approach is the application of QSAR to build ADMET (absorption, distribution, metabolism, elimination, and toxicity) models that can be used toward understanding the ADMET properties of lead compounds or drugs for optimization at the early stages of drug discovery [19,21,23,34,35,36]. These “global” QSAR models are diffuse and less interpretable in prediction. However, the global models address aspects of metabolic stability, drug-drug interaction and drug toxicity. The second approach has concentrated on the building of QSAR models for smaller series of molecules that are structurally related and pertain to specific individual CYPs. These models are more robust and are better at predicting the properties associated with ligand structural features and the electronic environment of the binding cavities. The QSAR efforts in the second approach aim toward improving the efficacy of the inhibitors/substrates of the individual CYPs through knowledge-based design. This review will focus on the QSAR models that have been built for the series of inhibitors for individual CYPs. An attempt will be made to coalesce and present the knowledge derived from the many different models for each individual CYP. This review aspires to provide the reader with clear indications of the necessary and optimum characteristics of the inhibitors for individual CYPs.

## 2. QSAR Models for CYP Enzymes

### 2.1. CYP1A1

CYP1A1 is found in lungs, lymphocytes, placenta, and skin with low constitutive expression in liver and has been implicated in cancers caused by polycyclic aromatic hydrocarbons (PAHs). PAHs are the main substrates for metabolism by CYP1A1. Geneste’s group built QSAR models for the metabolism of 32 diverse compounds of the PAH series by CYP1A1 using multilinear regression analysis and compared it with a nonlinear statistical analysis using an artificial neural network [37]. The metabolic activities of the PAHs taken for this study were investigated by Shimada *et al*. [38]. Docking studies were followed by generation of energy descriptors computed during the docking process which were used for the subsequent QSAR analysis. They found three main descriptors: (1) the HOMO energy (energy of the highest occupied molecular orbital) which is directly linked to the ionization potential of the molecule; (2) hydrogen bond acceptor atoms in the ligand; and (3) “-PMF04” scoring function obtained from the docking studies correlated with the inhibition potency. The predictability of the linear model was improved by using neural methods. Using the same three descriptors, the artificial neural networking (ANN) model gave an *r^2^* value of 0.66 after 200 iterations. The ANN method illustrated better prediction for CYP1A1 metabolic property than the linear model.

Flavonoids are important dietary constituents and are known to induce or inhibit several CYPs such as CYPs 1A1, 1A2, 1B1, 2C9, 3A4, and 3A5. Flavonoids are known to act as inhibitors of CYP1A1 and CYP1A2 resulting in prevention of cancer by environmental carcinogens [39]. Iori *et al*. developed QSAR models for three series of flavonoids that included several naturally occurring flavonoids [40]. Two types of calculations were utilized to obtain the descriptors for the QSAR studies; (1) Quantum mechanical calculations on inhibitors involving isolated molecules in water and low dielectric solvents; and (2) molecular mechanical calculations on compounds in inhibitor-enzyme complexes. The descriptors that were mainly focused upon were (1) the CHARMM total interaction energy between the ligand and the enzyme (IE_tot_) per unit surface of the ligand (IE/surf); (2) the CHARMM total interaction energy (electrostatic) between the ligand and the heme; (3) the CHARMM hydrogen bonding contribution to IE_tot_; (4) hydrogen bonding acceptor complimentarity between the enzyme and the ligand; and (5) the ZINDO free energy of desolvation. For CYP1A1 inhibitory potency by the flavonoids, good correlation was found with the descriptors IE/surf, the hydrogen bond acceptor and the desolvation free energy. Additionally, they found that the most active compounds showed the most stable HOMO in water.

**Figure 1:**
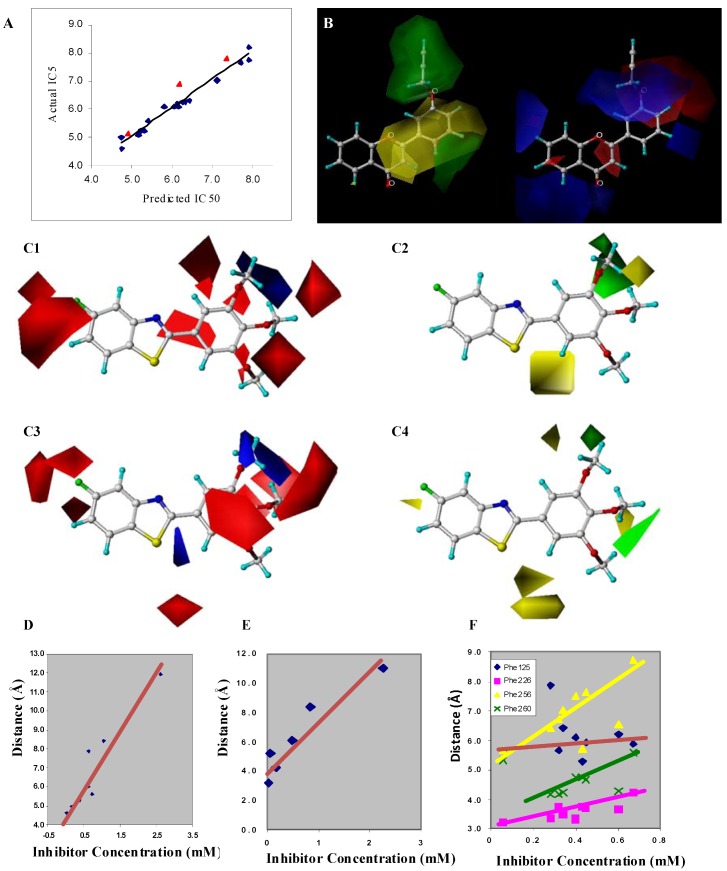
(**A**) Relationship between the actual and predicted logIC_50_ values for the CYP1A1. Data points representing the test compounds that were not included in the training set of the QSAR analysis are colored red; (**B**) CoMFA contour maps for the most active compound 3'-propargyloxy flavone against CYP1A1. Steric desirable and undesirable contours are colored green and yellow; +ve charge and −ve charge desirable contours are colored blue and red; (**C**) CoMFA contour maps with 5-fluoro-3',4',5'-trimethoxybenzothiazole, elecstrostatic and steric fields of Model 1 [(1) & (2)], and Model 2 [(3) & (4)]. (Figure adapted from reference [33]); (**D**) and (**F**) Relationship between the inhibitor concentration and heme-C distance for CYP enzymes 1A1, and 2B1; (**E**) Relationship between the inhibitor concentration and distance between centroids of aromatic rings of phenylalanine residues and inhibitor for CYP enzyme 1A2.

Methoxy and propargyl ether derivatives of hydroxyflavones and hydroxynaphthoflavones were synthesized in our laboratory in order to address potency, selectivity, and mechanism-based inhibition issues. The biological activities of the synthesized compounds were assayed for the CYPs 1A1, 1A2, 2B1, and 2A6 [41]. Most of these compounds were good inhibitors of CYPs 1A1 and 1A2, but did not inhibit 2B1 and 2A6. Comparative Molecular Field Analysis (CoMFA), a 3D-QSAR method, was used to understand the features of the inhibitors that contribute toward the biological activity against CYPs 1A1 and 1A2. The QSAR model built for CYP1A1 gave a cross-validated result of *q^2^* = 0.653 with five components and a conventional *r^2^* value of 0.982 with a standard error of 0.164. The electrostatic (29%) and steric (32%) descriptor components were the major contributors to this model with ClogP (18%) coming in as the next significant contributor (Figure 1A). The contour maps of the CoMFA model showed that the compounds with substituents in sterically favored region and electronegative atoms that fall in the region favoring negatively charged substituents showed the highest potency (Figure 1B). These results from the QSAR studies are being used to design new inhibitors that can be more potent and selective.

Benzoxazoles and benzothiazoles exhibit anticancer activities in sensitive cells and have been demonstrated to be CYP1A1 inhibitors [42]. The CoMFA method was used by Pan *et al*. [43] to build two models for the CYP1A1 inhibitors belonging to the benzoxazole and benzothiazole series of compounds (Figure 1C). Two types of alignment were used for building the CoMFA models: the minimum energy conformer-based alignment and the docked conformer-based alignment. The minimum energy conformer-based alignment gave the best model with a cross-validated *q^2^* value of 0.658 and a non-cross-validated *r^2^* value of 0.975. The electrostatic and steric descriptors contributed 61.3% and 38.7%, respectively, to the best model. The CoMFA contour maps showed that electronegative groups at positions 1, 2, 4 and 5 with less bulky groups at position 6 of the benzothiazoles would be beneficial for increasing the potency.

Lewis’ group performed multiple QSAR [18,19,35,44] and docking studies [44,45,46] on the substrates and inhibitors of several CYPs including CYP1A1. All of the QSAR studies on CYP1A1 inhibitors/substrates have shown that the properties that influence the potency of the ligands are the most stable HOMO, planarity, π-π stacking, optimally placed acceptor and donor atoms, steric bulk, and desolvation (ClogP). Docking studies have revealed that the specificity for CYP1A1 can be achieved by targeting the CYP1A1 enzyme residues Y72, S84, G187, and D282 for interaction with the ligand [40]. The docking studies on arylacetylene inhibitors of CYP1A1 by our group demonstrated a direct relationship of the inhibition potency to the distance between the triple bond and the heme Fe (Figure 1D) [47].

### 2.2. CYP1A2

In humans, CYP1A2 is expressed in the liver (10% of total CYP content) and is responsible for the activation of aromatic and heterocyclic amines, PAHs, and numerous therapeutic drugs. Procarcinogen activation by CYP1A2 has one of the most serious effects on an individual’s susceptibility to cancer. Inhibition of CYP1A2 can have major implications on cancer prevention. Indeed the cancer preventive effect of naturally occurring flavonoids is attributed to their interactions with CYP1A enzymes. Roy and Roy [48] preformed QSAR analyses on 21 naturally occurring flavonoids that inhibit CYP1A2 using 2D and 3D descriptors. An array of chemometric tools that include stepwise multiple linear regression (MLR), partial least squares (PLS), genetic function approximation (GFA) and genetic partial least squares (G/PLS) were used for the QSAR analyses. A K-means clustering technique with 2D descriptors addressing topological, physiochemical, and structural indices coupled with 3D descriptors that deal with spatial indices were used for the QSAR analyses. The G/PLS model with 2D descriptors was deemed the best model based on its highest *q^2^* value of 0.683 and *r^2^* value of 0.840. The 2D descriptors Balaban JX, kappa shape *^3^Kα*, connectivity indices and E-state indices, 3D-descriptors Jurs parameters and shadow indices were found to have maximum impact on the QSAR models. These studies showed that the presence of hydroxyl groups at 5 and 7 positions of the benzopyran-4-one nucleus is essential for the enzyme inhibition potency. In addition to the QSAR studies on flavonoids’ inhibition of CYP1A1 given in the earlier section, Iori *et al*. performed similar studies on CYP1A2 inhibition by the flavonoids [40]. It was found that interaction energy per unit surface of the ligand (IE/surf) descriptor explained 89% of the variance of the flavonoids inhibitory potency for CYP1A2. The most potent flavonoid inhibitors of CYP1A2 showed the most stable HOMO in water. The 3D-QSAR model (using CoMFA methods) developed in our laboratory [41] for the methoxy flavone, hydroxyl flavone, and flavone propargyl ethers correlated to their CYP1A2 inhibition potency with a cross-validated *q^2^* value of 0.648 (two components), and a conventional *r^2^* value of 0.893 with a standard error of 0.177 for the CYP1A2. The ClogP descriptor contributed more than half (54.1%) of the coefficient for the QSAR model, with the other descriptors COMFA steric, COMFA electrostatic, and molecular weight contributing 12.2%, 19.0% and 14.6%, respectively.

**Figure 2:**
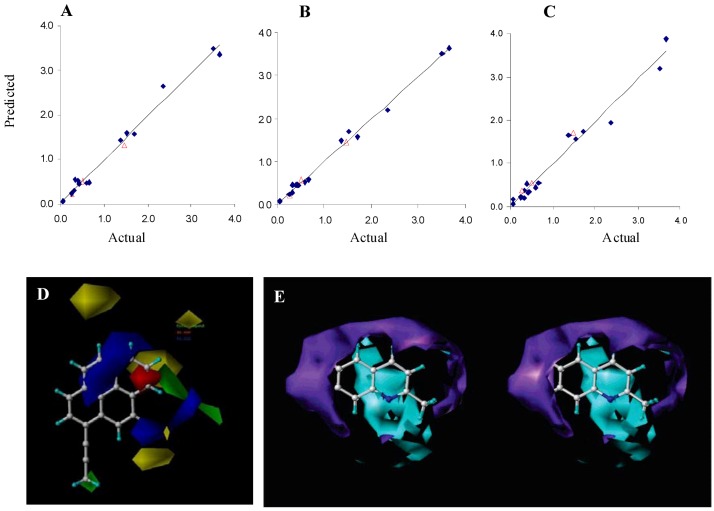
Relationship between predicted IC_50_ (mM) from CoMFA (**A**), CoMSIA (**B**) and HQSAR (**C**) models and the experimental IC_50_ (mM) values of CYP enzyme 1A2 inhibitors. Data points representing testing compounds that were not included in the training set of QSAR analyses are colored red; (**D**) CoMSIA contour map for the most active compound 1-propynylpyrene; (**E**) Stereofigure of GOLPE coefficient map with 2,7-dimethylquinoline as the reference structure. The cyan regions describe favorable interactions and the purple regions describe unfavorable interactions between the hydroxyl probe and the molecules (Figure adapted from reference [41]).

An in-house database of CYP1A2 inhibitors developed in our laboratory comprised of 36 compounds containing an acetylenic substitution in the form of a propargyl or ethynyl group of varied core structures (pyrene, phenanthrene, naphthoflavone, and naphthyl core structures) was used to build 2D- and 3D-QSAR models for the enzyme CYP1A2 [49]. Two alignment databases were obtained comprising of 19 and 34 molecules that were aligned using naphthalene and benzene substructures, respectively. QSAR analyses were performed on the two aligned databases using Comparative Molecular Field Analysis (CoMFA) (Figure 2A), Comparative Molecular Similarity Analysis (CoMSIA) (Figure 2B) and Hologram QSAR (HQSAR) (Figure 2C) methods. The models based on the first database yielded the best models with a CoMFA cross validation value *q^2^* of 0.667 and a Pearson correlation coefficient *r^2^* of 0.976; a CoMSIA *q^2^* value of 0.616 and *r^2^* value of 0.985; and a HQSAR *q^2^* value of 0.652 and *r^2^* value of 0.917. A second model incorporating 34 molecules aligned using the benzene substructure yielded an acceptable CoMFA model with *q^2^* value of 0.5 and *r^2^* value of 0.991. The CoMFA and CoMSIA analyses for the first databases proved to be better robust models than the HQSAR model. Nevertheless, the 2D fingerprint generated from the HQSAR model could be used for 2D database searches toward finding new CYP1A2 inhibitors. For the first alignment database, the descriptors that contributed to the CoMFA model were steric field (39.3%), the electrostatic descriptors (30.1%), and the ClogP descriptors (30.6%); the descriptors that contributed to the CoMSIA model were steric (7.0%), electrostatic (29.3%), hydrophobic (10.8%), hydrogen-bond acceptor (8.2%), hydrogen-bond donor + acceptor (steric, 8.2%), steric + electrostatic (steric, 7.0%) and steric + electrostatic (electrostatic, 29.3%). Comparison of the contour maps from CoMFA and CoMSIA showed high level of compatibility with the active site of CYP1A2 thereby reinforcing our confidence in the models developed for the first database alignment (Figure 2D).

Fifty two inhibitors of CYP1A2 that included derivatives of naphthalene, lactone and quinoline were subjected to 3D-QSAR studies by Korhonen *et al*. [50] using CoMFA and GRID/GOLPE analysis (Figure 2E). It was found that hydrophobic interactions played an important role in inhibitor binding potency for CYP1A2. The CoMFA contour maps depicted a large steric-favored region that corresponded to the presence of mostly hydrophobic and aromatic residues in the binding pocket of CYP1A2. The GRID/GOLPE analysis was in agreement with the CoMFA analysis with respect to H-bonds and electrostatic fields and gave insight into the shape of the interaction regions. This model gave the best predictions for biphenyls and certain dichloronaphthalenes. The model was also successfully used to predict the CYP1A2 pIC_50_ values of well known inhibitors of other CYP enzymes.

Various machine learning techniques, such as Associative Neural Networks (ASNN), Kappa Nearest Neighbor (kNN), Random Tree (RT), Random Forest (RF) and decision tree methods have been used to develop models for a large set of CYP1A2 inhibitors (400 to ~8,000 compounds) based on fragment-based descriptors, 2D topological descriptors and sets of 0D to 3D descriptors (Dragon, E-state and ISIDA SMF) [51,52]. The ASNN model in combination with the full descriptor set gave the best accuracy of 83% correct classification. The most accurate models employed Dragon 3D descriptors indicating the importance of 3D information in predicting CYP1A2 inhibition activity. 

Molecular planarity (area/depth^2^ ratio) has been shown to be an important attribute for the inhibitors of CYP1A2. Lewis *et al*. found that relative molecular mass in addition to molecular planarity was able to explain potency variation for a series of 23 compounds with diverse structures but possessing planarity [18]. Docking studies by our group [47], for the polycyclic aromatic hydrocarbons that are mechanism-based inhibitors of CYP1A2 showed that the π-π interactions with the four phenylalanine residues play a more dominating role in determining the potency of the inhibitors (Figure 1E). Additionally, favorable positioning of the hydrophobic groups of the inhibitors’ side chains in the hydrophobic region of the binding pocket promoted the potency of inhibition. Interactions with the residues V187, N272, R68, and T458 of CYP1A2 could improve selectivity for this enzyme [40]. 

### 2.3. CYP1B1

CYP1B1 is a unique enzyme that is expressed in many tumor types relative to normal tissue. CYP1B1 also has the ability to activate several carcinogens in the chemical classes of PAHs, heterocyclic/aromatic amines, and nitropolycyclic hydrocarbons. CYP1B1 catalyzes the hydroxylation of estradiol primarily at the C-4 position which could play an important role in estrogen-related tumorigenesis. Inhibition of CYP1B1 is an attractive strategy for chemoprevention. Gonzalez *et al*. [37] have developed QSAR models for PAHs and heterocyclic aromatic compounds as substrates of CYP1B1. A Multi-Linear Regression analysis (MLR) model was obtained with the LigandFit docking results which gave good predictivity. The HOMO electronic descriptor and the structural descriptor Molecular-SASA (Molecular-Solvent Accessible Surface Area) of the ligand were shown to be significant contributors to the model. This suggests that the favorable steric complementarity between the binding site and ligand geometry improves the potency. The docking scoring function Ludi1 that governs H-bond interactions between the ligand and the protein was also an important contributor to the QSAR model. Lewis *et al*. [18] found a good correlation between binding affinity and logP derived partitioning energy in the QSAR model for 12 compounds that inhibit CYP1B1. The model indicated the presence of two H-bonds and two π-π stacking interactions in the active site of CYP1B1. 

### 2.4. CYP2A6

**Figure 3:**
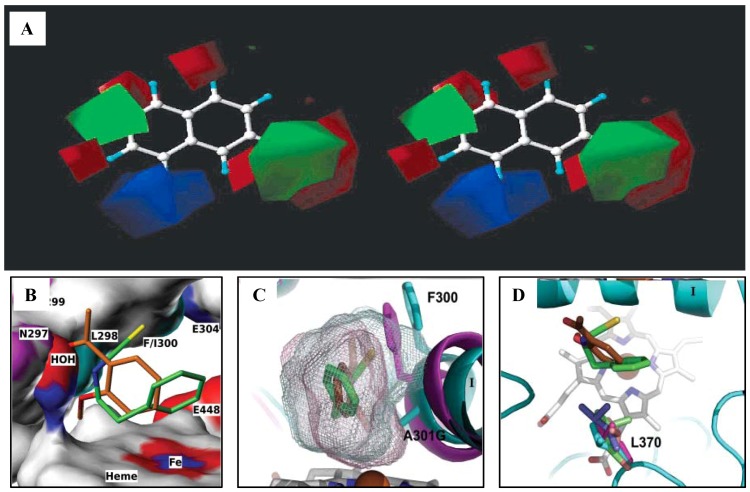
(**A**) Stereo figure of color contour maps of CYP2A6 CoMFA with the LUMO field. Areas where more negative partial charge and bulkier groups increase binding affinity are represented in red and green, respectively. Blue and yellow represent areas where more negative partial charge and bulkier groups decrease binding affinity, respectively. The reference structure is 2-fluoronaphthalene. (Figure adapted from the reference [45]); (**B**) Results of COMBINE analysis. Electrostatic features as gauged from COMBINE analysis are depicted for the high-affinity ligand (phenethyl isothiocyanate colored green bound to CYP2A13) and low-affinity ligand (2-methoxyacetophenone colored orange bound to CYP2A6). The protein surface is a composite of the highly conserved CYP2A6 and CYP2A13 structures, with features colored as follows: blue, favorable electropositive; red, favorable electronegative; cyan, unfavorable electropositive; magenta, unfavorable electronegative; (**C**) high- and low-affinity ligands-phenethyl isothiocyanate and methoxyacetophenone colored as in B, represented in the presence of steric features as gauged from COMBINE analysis represented with the CYP2A13 parental protein (cyan ribbons and mesh) and the CYP2A13 A301G mutant protein (magenta ribbons and mesh). Voids were calculated by VOIDOO; (**D**) conformational variation of Leu370 as a function of the CYP2A active site (CYP2A6, green; CYP2A13, cyan; CYP2A13/L366I, salmon; CYP2A13/G369S, blue; CYP2A13/H372R, magenta) with the bound ligands phenethyl isothiocyanate and methoxyacetophenone (colored as in B) are shown for reference. Figure (B), (C) and (D) have been adapted from reference [50].

The active human CYPs in the 2A subfamily are 2A6 and 2A13. CYP2A6 is expressed primarily in the liver and CYP2A13 is expressed primarily through the respiratory system. Both of these enzymes metabolize nicotine and nicotine-derived compounds cotinine and 4-(methylnitrosamino)-1-(3-pyridyl)-1-butanone. Both CYP2A6 and CYP2A13 have been implicated in tobacco-related lung cancers and the inhibition of these enzymes has been the area of active ongoing research worldwide. Several compounds were found to inhibit CYP2A6 *in vitro*. Rahnasto *et al*. developed two QSAR models (CoMFA and CoMSIA) based on the homology model of CYP2A6 [53]. The Leave-Some-Out (LSO) and Leave-One-Out (LOO) methods gave the best CoMFA models. Due to the stringency of the LSO model, this was chosen as the optimum model with four components. The CoMSIA model was a statistically poorer model. The contour maps of CoMFA and CoMSIA models provided insights for the design of a database search query. 10 compounds out of the search results were found to inhibit CYP2A6 with an IC_50_ of <10 μM. A new CoMFA model was created based on the inhibition data of the new set of CYP2A6 inhibitors and X-ray crystal structure of CYP2A6. The crystal structure based 3D-QSAR model showed that a hydrogen bond with N297 and van der Waals interactions between the hydrophobic amino acids and inhibitor are key determinants of inhibition potency. Rahnasto *et al*. developed a series of naphthalene derivatives as CYP2A6 inhibitors (Figure 3A) [54]. Naphthalene which is structurally close to coumarin, is a potent inhibitor of CYP2A6. Two CoMFA models were developed for this series of compounds. The first model used steric and electrostatic field descriptors and the second model used the Lowest Unoccupied Molecular Orbital (LUMO) field. The second model based on LUMO field was statistically the most significant. This model showed a clear dependency of the inhibition potency on the electrostatic interactions. A partial negative charge at positions 1, 2, and 5 as well as a positive charge at position 8 of naphthalene increased inhibition potency. Roy and Roy [55] performed QSAR analysis on the same series of naphthalene and non-naphthalene derivatives that were developed by Rahnasto *et al* [54] using different electronic (Apol, Dipole, HOMO, LUMO and Sr), spatial (radius of gyration, Jurs descriptors, Shadow indices, Area, PMI mag, Density, Vm), shape (DIFFV, Fo, NCOSV, COSV, ShapeRMS, SrVol) and thermodynamic (AlogP, AlogP98, Molref) descriptors related to structural information. K-means clustering, a non-hierarchical classification method was used to classify the compounds into clusters. Genetic Function Approximation (GFA) technique and Genetic Partial Least Squares (G/PLS) algorithm were used to develop the models. The GFA-derived model performed well for internal validation where as the G/PLS model performed better for external validation. Rahnasto *et al*. [56] recently developed a new CoMFA model based on docking alignment using 85 CYP2A6 inhibitors developed in their laboratory. The CoMFA contour map contained electrostatic fields near N297 that were associated with the presence of H-bonds between the inhibitor and the enzyme. The CoMFA model was used to create virtual screening query features. Five benzothiophene molecules selected from the search showed good inhibition potency for CYP2A6. Additionally, three of this series were found to be mechanism-based inhibitors of CYP2A6. Garaghani *et al*. [57] used molecular docking and molecular dynamics simulation studies to construct a structure-based QSAR model using the dataset of molecules developed by Rahnasto *et al*. [54]. MLR and Least Squares Support Vector Machine (LS-SVM) methods were used for developing the QSAR models. LS-SVM model proved to be the superior one and this model indicated that steric and electronic interactions affected the inhibition potency of these inhibitors.

A series of pyridine analogs developed by Denton *et al*. [58] as CYP2A6 inhibitors was used for building 3D-QSAR models by van Damme and Bultinck [59]. DFT properties (electron density, HOMO, LUMO and Fukui *f*¯ function) were calculated using Gaussian 03. The standard steric and electrostatic fields were collectively used with the DFT properties to determine a combined CoMFA model. This study yielded a model in which the orbital information was correlated with the binding affinity of the pyridine analogs. The Fukui *f¯* field information together with electron density descriptors provided a robust predictive model that was tested on an external test set. The DFT-based molecular fields indicated that the electronic interactions between the ligand and the enzyme CYP2A6 determined the inhibition potency. Docking studies indicated that the main influencing regions were the H-bond donating capacity of the nitrogen atom, and the aromatic interaction of the aromatic ring in an edge-to-face manner with an aromatic residue of the enzyme. 

DeVore *et al*. [60] performed receptor-based QSAR studies using the COMparative BINding Energy (COMBINE) approach to identify and quantify individual protein/ligand interactions that contribute to affinities (Figure 3B–D). Three ligands (coumarin, 2'-methoxyacetophenone and phenethyl isothiocyanate) were used in this study with CYP2A6 parent, CYP2A13 parent and CYP2A13 mutant enzymes. The set of COMBINE descriptors used for QSAR analysis consisted of van der Waals and electrostatic interaction terms between the ligands and each residue present in the protein of interest. Partial least squares fitting (Simca P program) was used to train the QSAR model which yielded a single model that was able to predict binding affinities for 32 of the 36 possible combinations of the three ligands with CYP2A6, CYP2A13, and the 10 different CYP2A13 mutants (*R^2^* = 0.87, *Q^2^*_LOO_ = 0.60, 3 components). Six residues at the active site N297, L298, F299, F300, E304, E448 and a water molecule that facilitated H-bond bridging with the conserved N297 side chain amide NH_2_ depicted the most important protein-ligand electrostatic interactions and van der Waals contacts. COMBINE identified differing amino acids at positions 300, 301, and 117 that were important steric contributors. These three residues had substantial effects on ligand binding and metabolism. 

The various QSAR studies and docking studies on CYP2A6 inhibitors and substrates have shown the importance of several factors influencing the potency of inhibition. The H-bond formation with N297 seems to be the most important factor along with a cation-π interaction with the heme moiety and lipophilicity of the active site [18,56,60]. N297, L370, and F480 also have significant steric roles in the binding of the ligand to the CYP2A6 enzymes. Most of the residues that contribute to electrostatic interactions with the ligands are conserved between CYP2A6 and CYP2A13 and thus may not show selectivity towards ligand binding. 

### 2.5. CYP2B Subfamily

The CYP2B subfamily of enzymes consist of the isoforms 2B1, 2B4, and 2B6 that have been studied for substrate metabolism by site-directed mutagenesis and some of these isoforms have been targeted for inhibitor development. The CYP2B subfamily is expressed at low levels in humans, but they crucially metabolize many foreign compounds in mammals including important drugs. Several QSAR studies on the substrates and inhibitors of CYP2B enzymes have been published. CYP2B substrates are generally non-planar with high lipophilicity, and capable of making hydrogen bonds with the enzyme active site residues. Lesigiarska *et al*. [61] performed QSAR and 3D-QSAR analysis on a series of xanthates that are inhibitors of CYP2B1. The molecules were aligned using Sybyl database alignment. CoMFA was performed using the PLS method with electrostatic and steric interaction energy descriptors, and structural energy descriptors such as HOMO, LUMO, I_POT, P_MIN, HEAT_F, MV, MW, and ClogP. Direct correlations were found between the inhibition potency and the HOMO and LUMO values, lipophilicity, molecular size, and molecular volume. A linear relationship between the alkyl chain length of the substituents and the biological and chemical activities of the xanthates was found wherein inactivation potency decreased with increasing size of the alkyl side chain. Lewis *et al*. [62] developed QSAR equations for a series of barbiturate derivatives and 7-alkoxycoumarins that bind to CYP2B1. A parabolic relationship was found between the logP and −logK_s_, where K_s_ is the spectroscopic binding constant implicating the dependence of inhibition potency to the lipophilicity of the compound. Our group performed docking studies on a series of CYP2B1 inhibitors comprised of planar polycyclic aromatic hydrocarbon containing terminal acetylenic substitutions [38]. The binding pocket of CYP2B1 had two phenylalanine residues Phe115 and Phe297 along with multiple hydrophobic residues and two polar residues. Phe297 made an edge-to-face interaction with the inhibitors whereas Phe115 made an edge-to-edge π interaction with the inhibitors. The distance between the heme Fe and the ligand external carbon of the triple bond varied from 3.2 Å to 11 Å and showed a linear correlation to the inhibition potency (Figure 1F).

**Figure 4:**
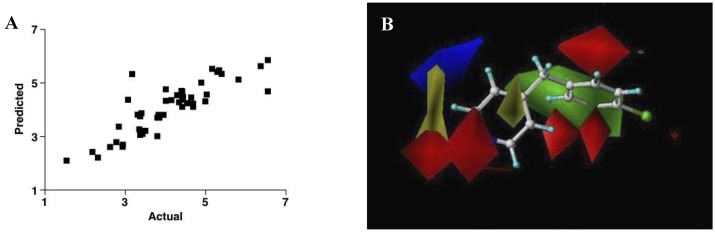
(**A**) Plot for the training set (actual/predicted IC_50_ values) of the CoMFA model; (**B**) Color contour maps of final CYP2B6 CoMFA model. Areas where more negative partial charge and bulkier groups favor an increase in inhibition potency are represented in red and green colors, respectively. Blue and yellow represent areas where more negative partial charge and bulkier groups favor a decrease in inhibition potency, respectively. The reference structure is 4-(4-chlorobenzyl)pyridine. Figure 4A,B were adapted from reference [53].

Korhonen *et al*. [63] examined the inhibition potencies of a structurally diverse set of compounds for the CYP2B6 enzyme. A QSAR model was built for the 41 compounds that showed inhibition of CYP2B6 (Figure 4A,B). 

The standard CoMFA electrostatic and steric fields were used for the PLS analysis. The model revealed the region that favored hydrophobic bulky groups and an acceptor atom that affects the inhibition potency. The QSAR studies by Lewis *et al*. on sets of diverse substrates revealed that hydrogen bonding is a key feature for the binding of substrates to CYP2B6 [19,62]. This feature is exemplified by the linear relationship between logP and −logK_m_ (K_m_ is the Michaelis constant for substrate metabolism). A similar analysis on the alkoxycoumarin substrates of CYP2B4 showed a linear relationship between the rate of dealkylation mediated by CYP2B4 and logP by itself and also when combined with HOMO energy [62]. Roy and Roy performed pharmacophore mapping, docking and QSAR studies on a diverse set of 48 compounds reported in the literature as CYP2B6 inhibitors [64]. For the generation of the QSAR models, the spatial (radius of gyration, Jurs descriptors, PMI-mag, density, Vm), thermodynamic (AlogP, AlogP98, molar refractivity), structural (MW, H-bond donor, H-bond acceptor, chiral centers, number of rotatable bonds) and topological descriptors were used. The models based on Genetic Function Approximation (GFA) were found to be better than the G/PLS-derived model. The GFA model with spline option was the best model with an *r^2^* = 0.774. Two important features were found for good inhibition activity-hydrogen bond acceptor and ring aromaticity with an ideal distance of 5.82 to 6.03 Å between these two features.

### 2.6. CYP2C9

**Figure 5:**
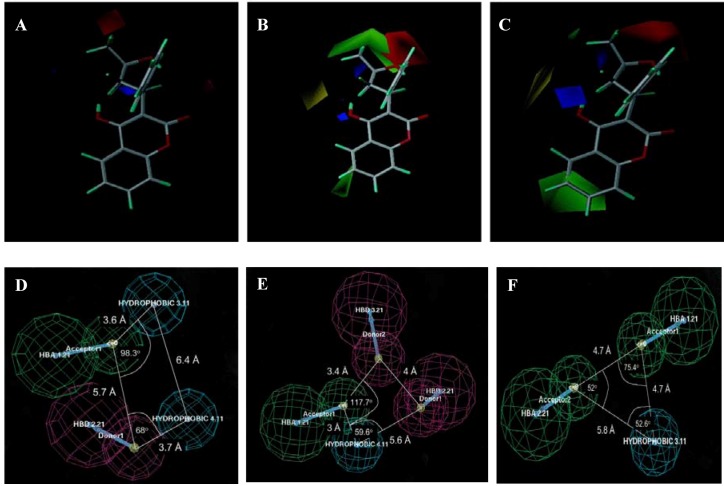
(**A**), (**B**) and (**C**): electrostatic and steric interactions in the original CYP2C9 CoMFA model, model 2C9 with 27 inhibitors and model 2C9 with 41 inhibitors. Red and blue indicate where more negative and positive charge will enhance binding. Yellow areas indicate where steric interactions hinder binding. Green areas depict where steric interactions enhance binding. Figures were adapted from references [54,55]. (**D**), (**E**) and (**F**) Catalyst three dimensional pharmacophore models 1, 2 and 3 with features illustrating hydrophobic areas (cyan), hydrogen bond donor (HBD, purple), and a hydrogen bond acceptor feature (HBA, green) with a vector in the direction of the putative hydrogen bond donor. These figures were adapted from reference [56].

CYP2C9 is expressed in the human liver to an extent of 15–20% of the total amount of CYPs. CYP2C9 is involved in the metabolism of drugs especially many of the commonly used polar acidic drugs in humans. CYP2C9 is competitively inhibited by non-steroidal anti-inflammatory drugs. Therefore, the need to evaluate drugs by QSAR for their ability to interact with CYP2C9 in their early stages of development is thought to be critical. Rao *et al*. initially built a QSAR model for CYP2C9 based on 27 inhibitors of (S)-warfarin metabolism *in vitro* that indicated the presence of an aromatic interaction site and two electrostatic interaction sites [65]. This QSAR model was later combined with a homology model followed by mutation studies to predict an important role for F114 as the aromatic interacting residue. This model was further modified by the addition of sulfonamides to the initial dataset to yield a more robust model with a dataset consisting of 41 molecules [66]. CoMFA methodology with standard defaults was used for determining the QSAR models by PLS analysis. The CoMFA model was placed in the active site of the homology model of CYP2C9. The model showed the region of aromatic interaction with F114, steric bulk-favored region near the I-helix and the *β*-5 sheet region close to the heme, anionic interaction site R105, and interaction of carbonyls of coumarins with D293 in the I-helix (Figure 5A–C). 

Ekins *et al.* constructed 3D- and 4D-QSAR models (Figure 5D–F) using the Catalyst and PLS approaches to Molecular Surface-Weighted Holistic Invariant Molecular (MS-WHIM) method. Three Catalyst models were built using the three datasets. These were: dataset1- nine structurally diverse compounds that are inhibitors of tolbutamide and diclofenac hydroxylations [67]; dataset2- 29 structurally similar CYP2C9 inhibitors of (S)-warfarin 7-hydroxylation [65]; and dataset3- two classes of structural analogs that are CYP2C9 inhibitors of tienilic acid hydroxylation and tolbutamide hydroxylation [68]. Model 1 had the lowest energy pharmacophore constructed by Catalyst and the features that affected the inhibition potency were two hydrophobes, one H-bond donor, and one H-bond acceptor. The lowest energy pharmacophore of model 2 showed that four features impact inhibition potency, namely, one hydrophobe, two H-bond donors, and one H-bond acceptor. In model 3, the three features that were deemed necessary for inhibition of CYP2C9 were one hydrophobe and two H-bond acceptors. Model 3 demonstrated a reasonable correlation of observed versus estimated IC_50_ (*r* = 0.71) and a higher total cost (59.9) than the null hypothesis (47.9). The general shape of all three Catalyst CYP2C9 pharmacophores were similar with distances between a H-bond acceptor and a second H-bond acceptor in the range 3.4 to 5.7 Å. Additionally, a hydrophobic feature was positioned 3 to 5.8 Å from a H-bond acceptor. The models from multiple datasets confirmed that multiple interaction determinants were required for inhibitors in the CYP2C9 active site. QSAR studies by Lewis *et al*. for 15 compounds including NSAIDS that were inhibitors/substrates of CYP2C9 showed that a linear relationship exists between lipophilicity and potency [69].

Jónsdóttir *et al*. used QSAR analysis to screen a large dataset of environmental chemicals for CYP2C9 activity [70]. They used an initial dataset consisting of drugs in clinical use and new drugs [71]. The QSAR model was developed with Leadscope Predictive Data Miner in which predefined structural features were used to mine the template library by structural analysis. Additionally descriptors such as AlogP, H-bond acceptors, H-bond donors, Lipinski score, atom count, MW, polar surface area, and rotatable bonds were used for model development. The number of optimal PLS factors for the model was optimized using 10-fold (10 times 10% leave-out) cross-validation. This model was used to screen a list of 51,067 chemicals from the EINECS list (European Inventory of Existing Commercial Chemical Substances). The QSAR model for CYP2C9 showed the selectivity towards slightly acidic compounds, large MW, large polar surface area, and larger number of H-bond acceptor atoms. The model predicted that ~30% of aromatic amines, PAHs and sulfonamides act as substrates/inhibitors of CYP2C9. It was found that flavones invariably acted as inhibitors of CYP2C9 [70]. 

### 2.7. CYP2C19, CYP2D6 and CYP2E1

CYPs 2C19, 2D6 and to a smaller extent 2E1 metabolize the majority of the known drugs. CYP2C subfamily overall plays a significant role in the Phase 1 metabolism of many drug substrates. CYP2D6 is one of the primary enzymes that metabolize compounds containing a basic amine. Lewis *et al*. have built QSAR models for various CYPs including CYP2C19, CYP2D6, and CYP2E1 [18,19,69]. Structural descriptors found by correlation analysis such as free energy of binding, molecular dipole moment, ratio of molecular length to width, lipophilicity, LUMO, and HOMO were used for the QSAR analysis. QSAR models were generated between the structural and biological variables which exhibit the best statistical fit with the data using Multiple Linear Regression analysis (MLR) method. The QSAR model for CYP2C19 showed that inhibitory potency for 10 compounds could be explained satisfactorily (*R* = 0.98) in terms of a combination between molecular mass, the number of H-bond donors and acceptors in the molecule, and lipophilicity character of the compounds. Most of these compounds made π-π stacking and H-bonds with the active site residues of CYP2C19. Rettie and coworkers synthesized derivatives of *N*-3 substituted nirvanol and phenobarbitol that were found to be potent and selective inhibitors of CYP2C19 [72]. The CoMFA models developed for these inhibitors indicated that stereochemistry was an important factor in determining inhibitor potency towards this enzyme and that CYP2C19 prefers to orient the *N*-3 substituents away from the active oxygen species [73]. Locuson *et al*. compared the CoMFA models derived by their group for benzbromarone derivatives that are inhibitors of CYP2C9 [74] with the above mentioned CoMFA model developed by Rettie’s group for CYP2C19 and found that CYP2C19 demonstrated greater stereoselectivity for the *C*-5 position of phenobarbitols than CYP2C9 [75]. Hydrophobicity of the *N*-3 substitution also contributed to the differences between the inhibition potencies for the two enzymes.

CYP2E1 is known to metabolize substrates with MW <100. The range of substrates includes solvents, fatty acids, industrial monomers, and other xenobiotics [24]. Due to the role of CYP2E1 in ethanol-induced hepatotoxicity and metabolism of procarcinogens, increased interest is seen in understanding the mechanistic aspects of this enzyme. Inhibition of aniline hydroxylation by primary alcohols for CYP2E1 demonstrated a correlation with the lipophilicity of the alcohol as indicated by its logP value [76]. Furthermore, Wang *et al*. showed that as the carbon chain length of alcohols and acids increased up to a length of 12 carbons, the *Ki* decreased [77]. Lewis *et al*. built QSAR models for the CYP2E1 inhibitors (10 selected inhibitors), that showed hydrophobic interactions specified by the lipophilicity parameters logP and logD7.4, exhibited a quadratic relationship to the inhibition potency which indicated that there would be an optimal value for compounds binding to the CYP2E1 enzyme [18,19]. The total number of H-bond acceptors and donors, active site π-π stacking, and relative mass also contributed to the model indicating that electronic effects are also in operation. 

The QSAR model developed by Lewis *et al*. [19] for substrates of CYP2D6 demonstrated the contributions from molecular mass, number of H-bonds, and active site π-π stacking interactions to the binding affinity. Vaz *et al*. developed a CoMSIA model for aryloxypropanolamine derivatives that were found to inhibit CYP2D6 [78]. The structural alignment was performed in Sybyl using the nitrogen, neighboring hydrogen, and the oxygen of the ethanolamine functionality. The steric and electrostatic descriptors were used for the CoMSIA analysis that was performed using the PLS method of analysis. The steric field contributed 51% toward the QSAR. Docking studies in addition to the QSAR studies showed that these molecules preferred to adopt a U-shaped conformation that is stabilized by π stacking with the active site aromatic residues. Two H-bonds were evidenced in the docking studies between the amino and hydroxyl groups of the inhibitor and the residues E216 and S304 of CYP2D6. Jónsdóttir *et al*. performed QSAR studies on CYP2D6 substrates/inhibitors (consisting of drugs in clinical use and new drugs) that used the methods similar to the one outlined earlier for CYP2C9 [70]. The resulting model was used to screen a list of 51,067 chemicals from EINECS. The model identified the importance of aromatic amines and other nitrogen containing compounds among CYP2D6 substrates/inhibitors. Carboxylic acids and oxycarbonyl groups were seen as the key negative feature for the substrates, but not for the inhibitors, implicating selectivity of CYP2D6 towards basic molecules that contain nitrogen atoms. CYP2D6 is known to be an important enzyme for the metabolism of aromatic amines. The model indeed identified 61% of aromatic amines in the EINECS database as CYP2D6 substrates and 18% as CYP2D6 inhibitors. Ai *et al*. used various methods such as CoMFA, CoMSIA, the Molecular Electrostatic Potential (MEP) and docking to investigate the inhibition of CYP2D6 by quinidines and quinines [79]. These studies revealed an interesting insight into the binding modes of these molecules. They found that quinidine blocks the entrance of the active site of CYP2D6 with its quinuclidine substructure, whereas quinine adopts an inverse binding pose which keeps the entrance open leading to the higher inhibition potency exhibited by quinidines. Hammann *et al*. employed machine learning methods (*k*-nearest neighbors, decision tree induction using the CHAID and CRT algorithms, random forests, ANN, and support vector machines using the Radial Basis Function (RBF) and homogeneous polynomials as kernel functions) to classify 335 structurally diverse compounds for their interaction with CYP1A2, CYP2D6, and CYP3A4 [80]. The best model indicated that CYP2D6-substrate interactions seemed to rely on ionizability and lipophilicity of the compound. This result was found to be in agreement with the observation that basic compounds interact with CYP2D6 via an ion-pair interaction with the aspartic acid residue in the active site.

### 2.8. CYP3A4

CYP3A4 is responsible for more than 50% of drug metabolism. It is a broad specificity oxygenase which is capable of metabolizing compounds belonging to diverse structural classes of drugs potentially leading to drug-drug interactions. Therefore, the development of CYP3A4 inhibitors has been intensely pursued. The notable feature of CYP3A4 is its active site which is considerably larger than that of any other CYP isoform. Didziapetris *et al*. developed a Structure Activity Relationship (SAR) model using GALAS (Global, Adjusted Locally According to Similarity) modeling method [81]. This method uses a combination of two approaches: a global model for the prediction of the property of interest, and a similarity-based local correction model. The literature dataset collected from various literature sources and the PubChem dataset from NCBI were used. The first step of the GALAS model is a logistic PLS with predefined set of fragments as independent variables, a baseline model of CYP3A4 inhibition which is linear and additive. The model showed that a strong basic group or an acidic group in the compound reduces the probability for a compound to inhibit CYP3A4. An increase in the size of the molecule with the incorporation of hydrophobic aliphatic or aromatic residues results in a higher probability for the compound to inhibit CYP3A4. The model also highlighted the importance of MW and lipophilicity on the inhibition potency of compounds for CYP3A4. In addition, the model showed dependence on the presence of hydrophobic groups which is also seen in the crystal structure of the enzyme in which a cluster of phenylalanine residues are seen in the active site. This model was found to be trainable to the needs of projects with new structural classes, with data from an assay with a different potency threshold and with data from similar assays. 

Roy and Roy developed QSAR models using 2D and 3D descriptors for a series of 36 aryloxypropanolamine compounds using the statistical techniques Genetic Function Approximation (GFA) and Genetic Partial Least Squares (G/PLS) [82]. The G/PLS model obtained from MFA with common substructure search and maximum common subgroup alignments were found to be the best model. Molecular Field Analysis (MFA) showed the importance of U shape conformation of the substrate for optimal inhibitory activity on CYP3A4. Roy and Roy developed another QSAR model for a database of 28 structurally diverse CYP3A4 inhibitors using the statistical tools of linear methods [Multiple Linear Regression with FactorA as preprocessing step (FA-MLR), stepwise MLR, PLS, GFA, G/PLS] and non-linear methods [ANN] [83]. The G/PLS model appeared to be the best predictive model with an *r*^2^ value of 0.916. The logP descriptor appeared to be the most important factor affecting the inhibition potency of CYP3A4 inhibitors. 

Mao’s group used Multiple Pharmacophore analysis (MPH) to develop a QSAR model as a conceptual extension of the traditional QSAR modeling by the Cerep software (the Bioprint Program Manager) wherein multiple pharmacophores are recognized [84]. A large dataset of 2,400 marketed drugs were used for this study. They found that MPH provided the basis for comparative binding using pairwise comparison of substrate IC_50_ activity values for CYP3A4. They succeeded in categorizing the substrates on the basis of the proximal and distal binding which provided a structural insight into their interaction with CYP3A4. Lewis *et al*. performed QSAR analysis on two sets of CYP3A4 substrates (statins and structurally diverse compounds). The models showed that lipophilicity relationships were evident which could be rationalized in terms of typical active site interactions such as H-bonding and π-π stacking. The large size of the active site in CYP3A4 could lead to variations in gradients due to multiple binding sites in the heme environment which would be in accord with experimental observations.

## 3. Conclusions

Multiple methods of QSAR analysis that have been performed for different CYP enzymes have been explored in this review. The results and conclusions from these QSAR analyses are providing important insights into the nature of the compounds that can act as inhibitors of the individual CYP enzymes. Due to the extensive role played by the CYPs in the metabolism of exogenous and endogenous compounds, the development of interacting compounds that act as inhibitors or prodrugs is proving to be an attractive proposition for the prevention and/or treatment of many human diseases. The advent of new algorithms in the near future will empower the QSAR analysis methods further which could lead to the availability of more information about the active sites of the CYP enzymes and the features affecting the potency/selectivity of ligands. The design and development of potent and selective inhibitors for individual CYP enzymes seems to be an achievable goal.
